# The Book World of Medicine and Science

**Published:** 1895-03-16

**Authors:** 


### The Book World of Medicine and Science.

The Prevention of Epidemics and the Construction and Management of Isolation Hospitals. By Roger M'Neill, M.D. (London: J. and A. Churchill. 1894 pp. 247. Price 10s. 6d.)

This is a useful book on a subject of wide importance. For those who are actually engaged in the planning and construction of hospitals it will not take the place of Burdett's great work, but for that very large public, consisting partly of medical officers of health and partly of members of local boards and other sanitary authorities, to whom a knowledge of modern views and modern methods for the prevention of epidemics is essential, this book will admirably serve its purpose. The inter-relation between town and country is strongly insisted upon, and it is shown that while the larger towns are gradually equipping themselves with means for coping with epidemics, the very poor provision hitherto made for this purpose in country places renders them a constant source of danger to the towns with which they have communication. The presence of infectious disease among the poor is also shown to be a danger to the rich; the repression of such diseases is, in fact, a matter for the whole community to deal with; and it is urged that compulsory rates for the purpose are a good investment. To get the full benefit, however, of this investment it is necessary that it should be so used that isolation shall be possible from the very beginning of an epidemic; in fact, the isolation of first cases is the very key to success, and therefore the hospital should exist ready for immediate use in every district. As the author says, "The expense of erecting and maintaining isolation hospitals may at first sight appear to be out of proportion to the small number of patients *treated* in them. The primary object of providing isolation hospitals is, however, to *prevent infection from spreading*." The value of the hospital must be measured more by the cases it prevents than by those it treats. Details are given in the book as to the expense of erecting and maintaining hospitals; the number of beds which should be provided per 1,000 of population; the distance to which patients can properly be conveyed; the proper size of the site for such fever hospitals; and various questions in hospital construction are entered into more or less fully. The very important recommendation is made that for hospital purposes, small boroughs should join with the surrounding rural districts. "Under the present system counties in Scotland are divided into districts; each district is managed by a separate local authority, or district committee, almost independent; and, dotted here and there in the districts, burghs are to be found entirely independent of county councils. If one or more of these districts or burghs do not provide sufficient means to prevent the spread of disease, the surrounding area will be continually infected by them." Some very careful remarks are made under the head of disinfection, and it is shown how, by comparatively cheap and simple methods, thorough disinfection of all articles used in a hospital may be carried out. All soiled linen is to be placed at once in a steeping tank, containing a solution of corrosive sublimate (1-1,000), and should subsequently be boiled. The object of the soaking is more to prevent the scattering of dust or volatile products than to act as a disinfectant, the sublimate is to disinfect the liquid. For such articles as cannot be boiled, an apparatus should be supplied in which they can be exposed to a current of steam, and it is pointed out that this apparatus need not be of an expensive character, as pressure is not necessary. Sufficient steam must, however, be supplied to blow off at 212 degrees after passing through the clothes; of course, these will come out damp, but that is a minor evil. It is much cheaper, where an apparatus is only occasionally used, to dry the clothes than to provide a high pressure apparatus. In an appendix, plans and elevations are given of various isolation hospitals, including those recommended by the Local Government Board.

